# Adolescents’ lived experience of panic disorder: an interpretative phenomenological analysis

**DOI:** 10.1186/s40359-022-00849-x

**Published:** 2022-06-06

**Authors:** Holly J. Baker, Amelia Hollywood, Polly Waite

**Affiliations:** 1 School of Psychology and Clinical Language Sciences, University of Reading, Reading, RG6 6AL UK; 2 School of Pharmacy, University of Reading, 1.05b Harry Nursten Building, Whiteknights, Reading, RG6 6DZ UK; 3 Department of Experimental Psychology and Department of Psychiatry, University of Oxford, Radcliffe Observatory, Anna Watts Building, Woodstock Rd, Oxford, OX2 6GG UK

**Keywords:** Panic disorder, Adolescence, Youth, Lived experience, Qualitative, IPA

## Abstract

**Background:**

Panic disorder is a debilitating anxiety disorder that has a serious impact on adolescents’ social and academic functioning and general wellbeing. Panic disorder is experienced by around 1 to 3% of the adolescent population. The aim of this study was to examine adolescents’ experiences of having panic disorder.

**Methods:**

Semi-structured interviews were conducted with eight adolescents with a primary diagnosis of panic disorder. Interpretative Phenomenological Analysis was used to gain an understanding of adolescents’ lived experience of panic disorder.

**Results:**

Two superordinate themes were identified: (1) Drowning in sensations, and (2) An unacceptable self. The findings show that adolescents experience panic disorder as extremely overwhelming and unpleasant, with debilitating feelings of drowning in sensations. Adolescents’ experiences largely fit with the cognitive model of panic, in which catastrophic misinterpretation of bodily sensations is associated with anxiety, avoidance, and safety behaviours, creating a vicious cycle. Attempts to avoid or prevent the attacks appear to inadvertently make them worse. Social worries, feeling broadly misunderstood, and unhelpful responses from others, contributed to feelings of being different or abnormal and were connected to a negative self-concept. Negative social interactions with teachers and peers in the school environment were particularly damaging.

**Conclusions:**

These findings offer new insight into these adolescents’ lived experience of panic disorder and highlight the need for adolescents to access timely, evidence-based treatment, as well as the need for increased awareness and understanding of panic disorder in schools.

**Supplementary Information:**

The online version contains supplementary material available at 10.1186/s40359-022-00849-x.

## Introduction

Panic disorder is a debilitating anxiety disorder, characterised by repeated, unexpected panic attacks, involving physical symptoms, such as a racing heart, dizziness and chest pain, along with a fear of recurring attacks and changes in behaviour to avoid further attacks [1]. Less than 0.5% of pre-adolescent children (aged under twelve years) experience panic disorder [1, 2]. However, panic disorder is experienced by around 1 to 3% of adolescents [3–5], with peak onset between 15 and 19 years of age [6]. Panic disorder commonly co-occurs with other anxiety disorders, particularly agoraphobia [7] and is more prevalent among girls (1.7%) than boys (0.5%) [5]. It typically has a negative impact across different areas of adolescents’ lives, including social interactions and academic functioning [8]. Recent evidence suggests that clinicians appear to commonly have difficulty identifying panic disorder in adolescents [9]. While there are cognitive behavioural treatments that have been demonstrated to be effective in the treatment of panic disorder in adolescents [10], a significant minority continue to experience panic disorder post-treatment. Therefore, it is crucial that we develop a greater understanding of adolescents’ experience of panic disorder to improve its identification and treatment.

The existing literature gives some indication of the diagnostic symptoms experienced by children and adolescents with panic disorder. The most common symptoms experienced by more than two thirds of children and adolescents (aged 8–17 years) with panic disorder appear to be dizziness, shortness of breath [11], palpitations and shaking [12, 13]. Around half of young people also report experiencing depersonalisation/derealisation (i.e., feelings of unreality in terms of the self and others/surroundings), and cognitive symptoms, including fears of dying [11, 13, 14] and of going crazy or losing control [11, 13, 14]. This is consistent with evidence of an association between panic disorder and anxiety sensitivity [8, 15] i.e., a tendency to believe that the experience of anxiety causes illness, embarrassment, or further anxiety. Kearney, et al. [8] found that children and adolescents aged 8–17 years had higher levels of anxiety sensitivity than those with other anxiety disorders and Elkins, et al. [15] found a significant association between anxiety sensitivity and panic disorder symptom severity in adolescents aged 11–17 years. However, it is notable that many of the studies examining the phenomenology of panic disorder in young people were published more than 20 years ago. In addition, caution is warranted in relying on findings from studies that include children as well as adolescents, especially given the very low rates of panic disorder among pre-adolescent children [1, 2]. Given that panic disorder is largely seen in adolescents rather than children, further research is required specifically with adolescents, and to move beyond symptoms to better understand the broader lived experience of panic disorder in adolescents.

In contrast to adolescents, panic disorder in adults is well understood, with a well-validated model that allows us to understand the development and maintenance of panic disorder [16–18]. Clark’s cognitive model of panic [16] proposes that normal physiological anxiety responses are misinterpreted in a catastrophic way (e.g., an increased heart rate may be misinterpreted as a heart attack), and that the individual perceives immediate danger, causing further apprehension and bodily sensations that culminate in a panic attack. Raffa et al. [19] found that 90% of adults with panic disorder had between one and four feared consequences during a panic attack, with the most common fears being embarrassment, death, fainting, going crazy, losing control and inability to cope/loss of independence [19]. Safety behaviours are protective behaviours aimed at avoiding or preventing perceived threats and are present across anxiety disorders [20]. Avoidance strategies and safety behaviours are commonly used, for example, avoiding situations or activities that are perceived to elicit panic attacks [1], and contribute to the maintenance of panic cognitions [21]. However, it is not known if adolescents’ experiences of panic disorder are similar to adults, in terms of how they make sense of the sensations and what they do to try to cope during panic attacks.

It is possible that adolescents’ experiences of panic disorder differ to that of adults due to developmental differences, as well as environmental factors. Adolescence is characterised by physical, behavioural, social and cognitive changes [22, 23], with the adolescent brain undergoing structural and functional changes [23]. Neurological development of the ‘social brain’ [24] is of particular importance during adolescence and is informed by perceptions of how others view us (e.g., other people think I’m weird) [25]. Coupled with these changes, social interactions become more complex and more important than among younger children [26]. Furthermore, adolescents experience increased self-awareness [27, 28] and a continuing development of self-concept, which is particularly influenced by peers [25]. Social interactions also have an increased impact on adolescents’ psychological wellbeing compared with children and adults [29], with negative social interactions and peer rejection leading to worsened mood, increased distress and anxiety [30]. These factors are likely to be salient for adolescents with panic disorder, and negative experiences and interactions are likely to be particularly impactful, due to a combination of these developmental sensitivities. In addition, adolescents are likely to be in full-time education and living at home with parents, and so it may be particularly important to understand the role played by others (i.e., school staff, peers, and family members) in adolescents’ experiences of having panic disorder.

Qualitative research methods are particularly useful for exploratory research questions in the field of mental health, where little is already known about the phenomenon [31]. To our knowledge, there are no published qualitative accounts of adolescent experiences of having panic disorder (or indeed anxiety disorders more broadly). There is one qualitative interview study of 14–18-year-olds who had experienced panic attacks within the last year (but had not been diagnostically assessed for panic disorder) [32]. Semi-structured interviews of ten adolescents (mean age 16.7 years) were analysed using Interpretative Phenomenological Analysis (IPA). Six themes were identified that included panic attacks feeling intense, overwhelming, out of control and like a battle within themselves. Mental images enhanced the intensity of panic. As a consequence, adolescents felt isolated, disconnected from others, and the attacks had a negative impact on identity [32]. Hewitt et al. concluded that further research with clinical samples of adolescents with a diagnosis of panic disorder would be important in addressing the limitation of potential diagnostic heterogeneity in their sample and enabling further understanding of this phenomenon.

The current study is the first to explore the lived experience of adolescents with a primary diagnosis of panic disorder. We focused on adolescents aged 11–18 years, as panic disorder is prevalent within this age range, and this age group share similar environments (e.g., likely to be living at home with family members and in full time compulsory education). Specifically, the research aimed to gain an in-depth understanding of adolescents’ lived experience of panic disorder.

## Methods

This was a qualitative, one-to-one, semi-structured interview design. Ethical approval for the study was obtained from the University of Reading Ethics Committee (REF: UREC 19/46) and through the NHS Research Ethics Committee (REF: 19/SC/0287).

### Methodology

Interpretative Phenomenological Analysis (IPA) [33] is an idiographic approach grounded in understanding individual experiences [34] and has utility in its clinical application and within wider theoretical contexts [35]. IPA was used to understand adolescents’ subjective experience of the phenomenon of panic disorder. The lead researcher (HB) approached the analysis from a phenomenological philosophical perspective.

### Participants

As is typical for IPA, we aimed for a homogenous sample. Participants were included in the study if they were aged 11–18 years, had a DSM-5 diagnosis of panic disorder [1], experienced at least one panic attack in the preceding month and did not have an autistic spectrum disorder, learning disabilities, suicidal intent, or recurrent or potentially life-limiting self-harm. Eight participants were interviewed for the study, and all had panic disorder as their primary anxiety disorder diagnosis. Their demographic and clinical characteristics are shown in Table 1. In terms of demographic backgrounds, participants were aged from 13 to 17 years of age and represent a relatively homogenous sample in terms of sex (with only one male participant and the remainder female), and ethnicity (with only one participant from a background other than White British).

**Table 1:** Participants’ demographic and clinical information

Participant	Age	Ethnicity	PDSS-C total score	Panic disorder CSR	Agoraphobia CSR	Social anxiety disorder CSR	Generalised anxiety disorder CSR	Separation anxiety disorder CSR
Mia	16–17	White British	4	4	–	–	–	–
Andrew	13–15	White British	8	7	7	4	–	–
Emma	13–15	White British	24	6	6	6	5	5
Olivia	13–15	White British	7	5	5	–	–	–
Eva	13–15	White British	10	5	5	–	5	–
Alexandra	16–17	White British	9	6	–	–	–	–
Azita	16–17	Other	24	7	7	4	–	–
Lilly	16–17	White British	10	6	–	5	–	–

*SAD* Social anxiety disorder, *GAD* Generalised anxiety disorder, *SepAD* Separation anxiety disorder, *CSR* Clinician severity rating on the anxiety disorders interview schedule [ADIS; scores range from the clinical cut-off of 4 (moderate) to 8 (very severely disabling/disturbing)], *PDSS* Panic disorder severity scale for children (PDSS-C includes seven items; each rated on a 0–4 scale (maximum score = 28), with a higher score indicating greater severity)

### Recruitment

Participants were recruited using purposive homogenous sampling. All participants had been recruited to a NIHR-funded feasibility study of the treatment of panic disorder in adolescents, being conducted within the NHS-commissioned Anxiety and Depression in Young People (AnDY) Research Clinic at The University of Reading. Participants were referred for treatment by primary and secondary care services, or recruited through local advertising (e.g., in schools, GP surgeries and on social media). Once referred for treatment, an assessment was conducted to determine whether they met diagnostic criteria for panic disorder (i.e., recurrent, unexpected panic attacks with four or more symptoms and persistent worries about future attacks or related changes in behaviour) [1] and were eligible for the trial. Interviews for this study took place between October 2019 and October 2020. The first five interviews took place before the COVID-19 pandemic and UK lockdown, which began on 16^th^ March 2020. Three interviews took place during the pandemic.

If participants were eligible and agreed to take part in the trial, they were then approached face to face and asked if they would like to participate in a qualitative interview about their experience of having panic disorder. Written informed consent was given by adolescents aged 16–18 years (or assent for adolescents under 16 years of age). Parents gave written informed consent for adolescents under 16 years of age. Parents also gave written informed consent for themselves to complete measures as part of the trial. Participants were the first eight sequentially to agree to participate. One (male) participant in the trial declined to be interviewed but did not provide a reason. Although there was little variability in terms of sex and ethnicity, this reflects the wider trial sample. The sample size was typical of IPA as analysis is based on detailed, in-depth examination of a small number of cases [33].

### Procedure

Data were collected using one to one, semi-structured interviews conducted by lead researcher HB, a female postgraduate researcher in psychology who had training and experience in qualitative research. The study was carried out as part of the lead researcher’s PhD. The interviewer had not met participants prior to them agreeing to take part in the interview and was not involved in the wider feasibility study. Interviews took place prior to the participants beginning treatment for panic disorder. They were conducted with only the researcher and the participant present, either at the clinic (n = 4), in the young person’s home (n = 3) or via video-conferencing software (n = 1) (due to restrictions due to the COVID-19 pandemic). Participants were reimbursed for the time taken to participate. At the start of each interview the researcher (HB) explained that the purpose was to gain an understanding of young peoples’ experiences of having panic disorder. Interviews were guided by broad, open questions based on an interview schedule (see Additional file 1: Fig. S1) that was developed in line with IPA methodology and recommendations [33]. It included questions covering the participant’s experience of having a panic attack, perceived causes, and how panic had affected their life. Each participant determined the flow of the interview, including the topics and the depth to which they were discussed. Gentle prompts were used to suggest topics that seemed to be of importance to the participant. Interviews were audio-recorded and ranged from 18 to 63 min duration (mean = 40 min). Recordings were transcribed verbatim by HB and the transcripts formed the raw data. NVivo 11 software was used to organise the data. Pseudonyms were allocated to participants to protect their identity.

### Measures

The Anxiety Disorders Interview Schedule (ADIS-C/P; [36]), and Kiddie Schedule for Affective Disorders and Schizophrenia (K-SADS; [37]) were used to determine diagnoses of panic disorder and co-morbid disorders. Panic symptom severity was assessed using the Panic Disorder Severity Scale for Children [38]. Further information on all measures is included in Additional file 1: Fig. S2.

### Analysis

Analysis was completed in a six-step process in line with the recommendations for using IPA [33, 39, 40]. Initial exploratory notes were made on each individual transcript of emerging themes, as well as commenting on participants explicit meanings, focusing on thoughts and experiences expressed and on linguistic points of interest, (e.g., pauses, words/phrases emphasised by the participant). The lead researcher (HB) made interpretations that went beyond explicit meanings to implied meaning, exploring the emotional responses of both the participant and researcher to gain an understanding of participants’ subjective experience. Initial emergent themes were developed into subordinate and superordinate themes by exploring connections and patterns between cases, examining similarities and differences between accounts, while remaining closely tied to participants’ accounts. The lead researcher engaged in a double hermeneutic process, as she tried to make sense of the participant’s experience, while the participant was also trying to make sense of their own experience. The researcher made notes in a reflective log throughout the process, recording thoughts, feelings and interpretations of possible meanings and connections to theoretical perspectives. This allowed the researcher (HB) to explore her responses, bring awareness to assumptions and biases and how these may affect the interviews and analysis. Therefore, this process aided the researcher in ‘bracketing off’ [33] these assumptions and biases. Emergent themes, subordinate and superordinate themes were discussed with AH, a health psychologist and PW, a clinical psychologist, who are both researchers with qualitative expertise. Researcher biases and assumptions were considered and included previous experience working in adult (HB, PW) or child and adolescent mental health services (PW), conducting research into young people’s mental health (HB, PW) and adult physical health (AH, HB, PW). Alternative interpretations were considered and discussed. Themes were re-analysed in an iterative process, ensuring that each participant’s experience was incorporated. Emergent themes were re-ordered before establishing final subordinate and superordinate themes with illustrative participant quotes identified.

### Rigour and study quality

The study was carried out in accordance with the quality guidelines for qualitative research [41], including the four principles of sensitivity to context, commitment and rigour, transparency and coherence, and impact and importance. Sensitivity to context was demonstrated through paying particular attention to the interview process, taking time to put participants at ease, using informal language, being aware of the participants’ sensitivities and of the power imbalance between interviewer and interviewee. This sensitivity continued throughout the analysis process. Commitment and rigour were addressed by recruiting a sample that was homogenous in terms of all participants having a primary diagnosis of panic disorder (assessed using the same gold standard assessment process), being of a similar in age, and recruited to a feasibility study within the same clinical service. To increase study rigour and transparency, an audit trail was kept throughout the study and analysis process documenting how themes were developed ideographically and across participants. To increase the trustworthiness of the analysis, we also followed guidance for quality and validity specifically in IPA [42, 43]. This involved building a picture of experience case by case before identifying similarities and differences across cases, use of a double hermeneutic to develop meaning around the individual’s lived experiences and constructing a compelling narrative.

## Results

Analysis resulted in two superordinate themes: (1) Drowning in sensations and (2) An unacceptable self. An overview of superordinate and subordinate themes are presented in Table 2. Together these themes identify how adolescents experience panic disorder and the most salient aspects of their lived experience.

**Table 2:** Superordinate and subordinate themes and prevalence within transcripts

Superordinate theme	Subordinate theme	Participant pseudonyms							
		Mia	Andrew	Emma	Olivia	Eva	Alexandra	Azita	Lilly
Drowning in sensation	“A vicious kind of circle”	*	*	*	*	*		*	*
	“A different mentality”	*	*	*	*		*	*	*
	Losing the battle	*	*	*	*	*	*	*	*
An unacceptable self	Under the social spotlight	*	*	*	*	*	*	*	*
	The unhelpful helpers	*	*	*	*	*	*	*	*
	The outsider	*	*	*	*	*	*	*	*

* marks where theme was present in transcript

### Drowning in sensations

This superordinate theme represents adolescents’ experiences of panic disorder as an intensely unpleasant physical, cognitive, and emotional experience. Each of these elements were interwoven, as adolescents tried to make sense of what was going on in their bodies and minds. This superordinate theme is presented in three interrelated subordinate themes: “A vicious kind of circle”, “A different mentality” and Losing the battle.

#### “A vicious kind of circle”

The onset of panic attacks associated with panic disorder were experienced by each of the adolescents as a series of sudden overwhelming physical sensations. These sensations were present in the lead-up to, and during panic attacks, and included feeling their heart pounding, feeling they could not breathe, sweating, shaking, stomach pains, tingling in parts of the body, and dizziness. These sensations were experienced as confusing, frightening, catastrophic and totally overwhelming, as adolescents struggled to understand what was happening to them.

It normally starts in your fingertips and your toes go all really tingly and then like it just starts spreading up your legs and stuff and then you can’t move at all cus once the tingling starts, after that you can’t feel anything and then um like when the dizziness, you’ll like stand up or something and then you just, it’s like a spinning sensation, also like unsteadiness and you feel like if you don’t hold onto something, you’re just going to fall. You can’t breathe properly and like erm, you get like quite snotty, so it kind of all just, you kind of just drown in everything. If it starts happening, then I’ll think that I’m going to have a panic attack and then that will make me panic. When you’re having a panic attack you just think like the worst of everything (Emma).

Emma describes the onslaught of a series of sensations that sweep through her body, with the image of “drowning in everything” representing her feelings of being totally immersed in physical sensations. As Emma experiences these sensations, worst-case scenario thoughts lead to further feelings of panic and thinking about panic attacks “makes” her panic. All adolescents experienced intense thoughts that were catastrophic in nature, that were associated with anxiety, increasing feelings of uncertainty and fear, and contributed to the escalation of attacks in a cyclical way.

I always catastrophise about it. I was worried that I’d feel nervous, then it kept on going round and round like a vicious kind of circle, and then I started to kind of get like, develop more worries for like my health, and like I was going to have a heart attack (Olivia).

Olivia demonstrates how she interprets the physical sensations as meaning that something is seriously physically wrong and how this “vicious kind of circle” escalates the symptoms and worries to the point where, in that moment, she believes her life is under threat. Other adolescents also reflected on how, during a panic attack, they believe that the worst could happen, and they could potentially die.

At first, I struggled to breathe and then I felt like I was going to faint on the floor, it was terrifying, and I was just panicking, and I just kept like trying to hold on to my mum and like squeeze her. I didn’t understand what was going on or why I felt that way, so I was, I was confused as well. I thought I would die (Azita).

Azita’s description of her very first panic attack demonstrates how the lack of understanding about what was happening to her increased feelings of terror, as her fears escalate from worrying about fainting, to worrying that she was going to die. An overwhelming, terrifying series of unexplained physical sensations lead Azita to fear the worst.

#### “A different mentality”

This subordinate theme expresses how participants felt as though they were in a different state of mind, feeling that the way their mind operated was different to when in a non-panic state. Feelings of unreality, as though in a dream state, were experienced, and this was disorienting.

You’re in a different mentality. You kind of go into this kind of response that’s not logical, it’s like if your logically thinking about something, then you can weigh out the pros and the cons and you can tell yourself ‘well no nothing bad is going to happen, you’re going to be fine’, but in a panic attack you’re already, you’re past that and you’re already at a stage where you will just kind of, your body is telling you to get out of there, like get out of that situation. (Olivia).

Olivia’s experience of her mind being “different” and “not logical” emphasises the experience of being in an altered state of mind. During panic attacks, the capacity to realistically appraise the situation is lost and she feels unable to change her responses, beyond the point of being able to calm herself down.

I sometimes feel like I’m not really there, that I’m kind of dreaming (Mia).

Mia’s extract illustrates how she feels mentally separated or disconnected from the experience as her body is overwhelmed with physical sensations and her mind races with thoughts about what is happening to her.

I feel like the walls are just getting closer and closer and stuff even though they’re not. It’s like in, like adventure shows or movies. It feels sort of erm, slightly unreal (Emma).

The metaphor of being in a movie, or an adventure show, highlights Emma’s feelings of unreality. Emma’s excerpt also illustrates how this experience is a frightening and suffocating one, as she feels like she is being compressed between walls that are closing in on her, and there is no escape.

#### Losing the battle

This subordinate theme illustrates how, despite attempts to avoid, prevent, or bring an end to the panic attacks, once underway, adolescents felt they were unable to escape the experience, defeated by physical sensations and catastrophic thoughts.

I just felt like I was trapped. I was trapped, I couldn’t breathe. I don’t really know how to describe it but it’s, it’s not nice, because you feel like you can’t get out (Lilly).

As panic symptoms escalate and she feels unable to breathe, Lilly experiences overwhelming feelings of being trapped. Most adolescents experienced similar feelings of being trapped and needing to escape during attacks, and this is further demonstrated by Olivia:

In a panic attack I think your mind goes into like kind of, you know, the sirens on an ambulance, that’s what it kind of does and it like, it goes into an emergency and its thinking well there’s a danger, and you, those kind of lights just start kind of flashing [hands make flashing gesture] and then like, and then your mind, the only thing that you’re trying to think is you have to get out of here, you have to run, you have to, you have to go somewhere else (Olivia).

The language that Olivia uses expresses the urgent need to escape and depicts this experience as the mind being in an emergency mode. Olivia describes her brain giving off warnings, like ambulance sirens, signalling an immediate danger, as the wave of panic approaches and takes over her. This response overwhelms Olivia, as the only thing that she is thinking is how she can get out, run, and get away.

During panic attacks, some adolescents used techniques to try and bring them to an end.

I've tried this breathing technique a friend taught me. Breathe in four breaths, hold for four, breathe out for six. Sometimes it will do absolutely nothing. Sometimes it will just make it worse. The feelings I’m getting have just been intensified. I’ve tried to calm it down *thousands* of times, but I just can’t get it to calm down. So, then it stresses me out even more, because I’ve just got to the point where I just let them just go. At the moment I’m just trying, I just try and ignore them at the moment because I, I know I can’t do anything to calm down at this point, but I try sometimes, it just makes them worse... So realistically, I’ve just given up trying to calm them down (Alexandra).

Alexandra experiences these attempts to end the panic attack as a futile cycle of trial and error of strategies. As her symptoms escalate, she is unable to gain control over them, and cannot calm herself down. This leads to feelings of resignation, as she realises that she cannot control or avoid the panic state and has “given up” trying to stop them. All adolescents expressed similar feelings of ultimately being defeated by the overwhelming sensations.

It’s just kind of being overwhelmed with all these feelings that you can’t really control or like hide in a way, like it just all kind of floods out of you and makes you feel really weak. It just makes me feel really weak like I can’t, I feel like I can’t walk properly, like I just feel really like, not capable of doing things (Eva).

Eva’s account describes how the experience of panic disorder feels like a flood, an uncontrollable force that cannot be prevented. She is powerless, physically drained, and weak as she loses the battle against her panic symptoms.

### An unacceptable self

This superordinate theme illustrates how adolescents felt that they were unacceptable to themselves and to others. All participants experienced intense social worries and fears of being judged in relation to their panic attacks and worried about having attacks in front of people. These social worries were experienced by adolescents both with and without comorbid social anxiety disorder. Adolescents experienced unhelpful responses from others that appeared to stem from a lack of understanding of panic disorder. Negative interactions, feeling misunderstood and being rejected, contributed to feelings of being unacceptable to others or to themselves. These feelings fed into an overarching negative self-concept, feeling they were not normal and were an outsider. This superordinate theme is expressed in three subordinate themes: Under the social spotlight, The unhelpful helpers and The outsider.

#### Under the social spotlight

This subordinate theme represents participants’ worries about being judged negatively by other people or being stigmatised in relation to their panic attacks. These social worries were part of the experience of panic disorder for all adolescents in this study.

I’m trying, I’m trying to hide what’s going on, but I can’t, because in the moment you want to scream, you can’t breathe, you’re freaking out, but you also don’t want anyone to stare at you or to realize what’s going on, because obviously they won’t understand what’s going on. That kind of makes it worse, because while you’re trying to manage a panic attack, you’re also trying to look like you’re not having one, because you don’t want people’s judgment and even afterwards, you’re embarrassed because everyone just saw you freak out (Azita).

Azita’s extract emphasises how she feels that the panic is something she needs to hide from other people. The overwhelming, frightening experience of the attack is coupled with anxiety and embarrassment about how others may view what is happening to her and the need to contain it. “Obviously they won’t understand” shows us how Azita feels alienated, that other people don’t understand her experience, and that she will be judged negatively. This extract demonstrates how these social worries feed into the panic cycle, increasing symptoms, and intensifying the unpleasantness of the overall experience, as worrying about the social implications intensifies everything and “makes it worse”.

When panic attacks occur in the school environment, where young people are constantly surrounded by others, fears around being judged are commonly part of adolescents’ lived reality of panic disorder.

I was around other people, so it just made it worse, and people were looking, which made it also worse. I was aware that people were watching (Lilly).

Lilly experiences feeling heightened awareness of other people watching as she has a panic attack, explaining that being around other people and being watched makes the experience worse for her.

It just brings attention to you, and you don’t want attention. I’m like kind of breathing quite heavily, that’s when other people start to notice and then if someone um just looks at me, or asks if I’m ok, then it’ll get faster, cus I know that people are starting to realise, and then I’ll start getting a few more [symptoms] (Emma).

Emma further illustrates how having a panic attack while around other people, intensifies her panic attack, increasing her symptoms. If people do notice and start to look at her, the attack gets “faster”, demonstrating the experience of the increasing intensity of panic attacks due to being observed.

#### The unhelpful helpers

This subordinate theme illustrates how adolescents felt that other people who should be there to help, often lacked understanding about their panic disorder.

I feel like they [teachers] didn't understand what was happening (Andrew).

Andrew highlights feeling that school staff did not understand what was happening to him during panic attacks. Several participants had experienced this lack of understanding in school, where teachers had responded in unhelpful ways. This lack of understanding led to responses that were unhelpful, and often made the experience of having a panic attack worse for them.

Sometimes people try and help, but it doesn’t help and then I get mad at them cus they’re telling me to do this thing, and I’m like, that doesn’t work, I’ve already tried. I know they’re trying to help me, but it doesn’t help. They [school staff] were saying ‘breathe in now’ and then like if I didn’t then they kept telling me that I’m not listening to them. It doesn’t help. They kept standing over me and stuff and like threatening to, they were like ‘if you don’t come now, we’ll do this’ and stuff and they kept asking me questions and I couldn’t respond, so they, they started calling me rude (Emma).

Emma expresses how, even though sometimes other people offered help her during panic attacks, their intervention made things worse. She felt misunderstood as teachers interpreted her lack of response to them as her being rude. She felt frustration at having “already tried” strategies to calm down, and repeated instructions from teachers to “breathe in”, which only contributed to feeling totally overwhelmed and misunderstood.

I had a teacher who didn’t really understand. I was starting to feel like a big panic in class, like it was coming up, I knew it was coming, I had to leave like it would just get worse and worse, so I put my hand up, I said, ‘can I leave for a second’ like trying to breathe, and she was like ‘for what?’ I was like ‘I just really need to step outside’ and she said ‘no’ and I was like, ‘please, I really need to step outside’, she said ‘no’. So, I just sat there, and it got so bad that lesson, and from there I had such a fear of that class and that one teacher, that I just I couldn’t go back (Azita).

This scenario demonstrates the lack of power Azita felt; while in the school environment, she did not have the autonomy to just leave the room. Her worries about being prevented from leaving the classroom in subsequent panic attacks contributed to a cycle of worry and avoidance. This culminated in Azita never returning to that teacher’s classes, with a negative impact on her education.

Unhelpful reactions could come from peers (including friends) as well as school staff.

They can make fun of it a little bit and [pause], you know they kind of laugh a little bit about it but when really, it’s actually serious. I just want, I’d rather if they didn’t make jokes about it, because it’s quite like, upsetting. When I told them about, that I was doing this research, and I said they gave me some money, they were like oh ‘I want to have panic attacks too, oh no! on no! I’m panicking! I’m panicking!’ and you know, kind of doing that, and I was like ‘well no, that’s not what a panic attack is like, that’s not what it is’. But they just don’t really understand what it’s like. I was thinking at the time, I was just like, oh they joke around, it’s just kind of what teenagers do. But after, I was thinking well that just kind of proves they don’t understand (Olivia).

Although Olivia has confided in her friends about her panic disorder and how she has received some money as a thank you for taking part in research, her friends appear to overlook her experience of having panic disorder and instead make a joke of it, implying perhaps that receiving some money would make it worth experiencing panic attacks. This leaves Olivia feels invalidated and upset.

#### The outsider

This subordinate theme represents how adolescents felt that panic disorder was a part of themselves that they did not like and did not want, a part that was unacceptable to themselves and to other people. This often left them feeling like the outsider. This feeling was emphasised by experiences where they felt stigmatised or rejected and negative interactions with others contributed to feeling that they were not ok, did not fit in and were different to other people. Overall, this contributed to a negative self-concept.

As soon as I told them, they started just saying, just not hanging around me. I think they just don’t, maybe they didn’t know how to deal with me, or they just thought of me differently or they just, I don’t know. I’m not them, I don’t know how they [pause], I try not to dwell on it too much. But it does upset me sometimes that they just ditched me. Part of me just doesn’t feel like I fit in (Alexandra).

Alexandra feels left out, rejected, and abandoned, and that other people’s perceptions of her changed due to her difficulties. The fact that others do not know how to “deal” with her, leads to her feeling that other people do not understand her or know how best to respond. This contributes to her feeling that this is a part of herself which other people cannot accept and that she does not fit in.

I tend to just leave it out, cus I feel like that’s just a different part of me that I don’t really want. I feel like they’re just seeing this person that’s not really like human, just got problems. I feel like I don’t really fit in, I can’t be normal, but it also feels a bit weird, because I’m not like all of my friends, I’m not like everyone in my class, yeah, a bit of an outsider (Eva).

Eva’s account illustrates feelings of shame as she “leaves out” her panic disorder when talking to other people. She feels that other people see her as “not really human”, and this encapsulates the feeling of being alien and different to other people. The idea that people only see her as someone who has got “problems” illustrates her feelings that people do not really see who she really is beyond her panic disorder. The impact of this on her self-concept is that she feels she is not normal and does not fit in with her friends and peers, contributing to feeling isolated and alone with her experiences. These feelings of negative self-concept were shared with almost all the participants.

Why am I not normal? Why can’t I be like everyone else? It kind of feels a bit lonely, cus you’re like, well kind of it’s, it’s quite like being left out, because you just kind of want to be like everyone else and just kind of be able to sit through these kind of things but like you can’t, because like you’re not like everyone else (Olivia).

Olivia illustrates here how she deeply wants to be like other people and feel that she fits in. There is a sense of not understanding why she is not like other people, and this saddens and frustrates her. For Olivia, having panic disorder means that she is not like others, and does not fit in, she is an outsider. She is unable to participate in the things that her friends can, because of her panic disorder. She feels left out and lonely, and that fundamentally she is “not normal”.

## Discussion

This study explored eight adolescents’ (aged 13 to 17 years) lived experience of panic disorder and is the first qualitative study examining adolescents who have been diagnosed with panic disorder. Two superordinate themes were identified, capturing adolescents’ experience of panic disorder. The first superordinate theme, Drowning in sensations, was presented in three subordinate themes: “A vicious kind of circle”, “A different mentality”, and Losing the battle. The second superordinate theme, An unacceptable self, was presented in three subordinate themes: Under the social spotlight, The unhelpful helpers and The outsider.

Although our findings identified that adolescents experienced cognitive factors and symptoms that fit with current understanding of adults and adolescents with panic disorder, we also identified aspects of the panic experience that were of particular importance for adolescents. In line with the previous literature on common symptoms among adolescents [11, 12], the first superordinate theme, Drowning in sensations, represented the experience of panic disorder as typically being characterised by catastrophic cognitions and misinterpretation of bodily sensations as reflecting impending physical or mental danger (e.g., dying or losing control). The experience of drowning in sensations incorporated feeling a loss of control of the body and the mind, and of being defeated by panic attacks. This theme corresponds with the findings identified in Hewitt et al.’s [32] study of adolescents (aged 15 to 18 years) with panic attacks who identified that feeling out of control during panic attacks was a dominant theme. Misinterpretation of physical sensations is entirely consistent with the cognitive model of panic in adults [16, 17, 44–46], and associations between anxiety sensitivity and panic disorder in children and adolescents [15, 47]. Also consistent with this model, adolescents appeared to engage in a range of safety behaviours [48, 49], intended to prevent their feared catastrophes from occurring, inadvertently maintaining a vicious cycle of thoughts, sensations and behaviours.

The second superordinate theme; An unacceptable self, highlighted important factors in adolescents’ experiences of panic disorder that appeared to reflect this being a unique developmental period. Unlike adults, adolescents very often lacked the autonomy to leave situations freely during panic attacks, for example being made to remain in classrooms in school. In many situations, this meant negative interactions with teachers, and often (possibly inadvertently) unhelpful behaviours from teachers and peers, which contributed to feeling broadly misunderstood and appeared to intensify the experience. These findings correspond with those of Hewitt et al., who found adolescents had encountered a lack of understanding about their panic attacks, negative interactions with teachers and peers [32].

Also identified in the second superordinate theme, and of particular importance for adolescents, was the experience of being ‘Under the social spotlight’, with heightened social anxieties around having panic attacks appearing to contribute to the panic cycle. It was interesting that they were experienced both by adolescents with, and without comorbid social anxiety disorder and pervaded before, during and after attacks. Although consistent with findings that adults experience embarrassment about panic attacks [32], for the adolescents in this study, these worries extended beyond fears of embarrassment to worries about being rejected by peers, being socially excluded and/or being treated differently. This culminated in a sense of being unacceptable to themselves and to others. Furthermore, worrying about having panic attacks in social spaces within school, led to increased avoidance of those situations and intensified or escalated the panic attack cycle for adolescents, contributing to the negative impact of panic overall. This aspect of the panic experience may be especially salient for adolescents. The importance and impact of social relationships for adolescents has been well documented [25, 27, 50] and our findings emphasise the importance of understanding the interplay between social cognition, negative social interactions, and the panic cycle, specifically for adolescents who are in a sensitive phase of social development.

In the second superordinate theme, we also identified that adolescents’ experiences of panic disorder contributed to feeling like an outsider and having a negative self-concept. Adolescents felt they were different or “not normal” compared with peers. These feelings were compounded by a lack of understanding and negative social interactions with other people in connection with their panic attacks. This included being bullied, socially isolated or being perceived as “rude” or difficult during attacks. Negative self-concept, self-hate and self-blame during adolescence are associated with anxiety and depression [51], therefore, experiencing anxiety in itself may add to negative self-concept that is associated with additional worries, forming another kind of vicious cycle for these adolescents. Our findings in relation to self-concept also correspond with those of Hewitt et al., where adolescents’ identities were affected in a negative way [32]. Adolescence is a critical time for the development of a socially integrated self-concept, which is informed by perceptions of how others view us (e.g., other people think I’m weird) [25]. Therefore, these negative social experiences may be particularly impactful for adolescents with panic disorder.

Our findings have several clear implications. The overwhelmingly unpleasant and distressing experience of panic disorder and the negative impact on important parts of adolescents’ lives, such as their education, highlights the importance of being able to access effective, timely treatments. Our findings broadly support a cognitive conceptualisation of the disorder and fit with the current evidence base for the treatment of panic disorder in young people, which involves cognitive behaviour therapy [10, 38]. Given the prominence of avoidance and safety behaviours exhibited among the adolescents in this study, addressing these factors in treatment through exposure is likely to be vital [52]. It is also likely to be important that therapists liaise with school staff during treatment to ensure that teachers have guidance about how best to respond when the young person experiences panic attacks at school. The adolescents in this study reported associated social worries and negative self-evaluations; although successfully treating panic disorder may have a positive impact in these areas, further investigation of this will be important.

More broadly, our findings highlight a need for increased awareness and understanding among young people and school staff, so that adolescents experiencing panic attacks within the school environment are met with greater understanding from peers and can access appropriate help and support from staff. Mental health education has been demonstrated to reduce stigma and increase knowledge among school staff [53] and students [54, 55]. Therefore, providing psychoeducation, that includes information about dealing with anxiety and panic attacks through teacher training and the school curriculum (e.g., through personal social health and economic education lessons) is potentially a feasible and effective way to increase awareness and understanding among staff and students.

### Strengths and limitations

This research needs to be considered in light of several strengths and limitations. Our findings build on those of Hewitt et al. [32] by focusing on adolescents with a diagnosis of panic disorder, as opposed to young people who had experienced panic attacks, which can be associated with a range of psychological difficulties, and by including younger adolescents (aged 11–14 years) in the sample, as well as those aged 15–18 years. As is desirable in IPA, the sample was homogenous; all participants had a primary diagnosis of panic disorder (assessed through a gold standard assessment), were within the adolescent age range, had been referred for treatment, and were recruited through the same clinical service. We followed quality guidelines for qualitative research generally [41] and within IPA specifically [33, 42, 43] throughout the study. The lead researcher (HB) engaged in a double hermeneutic process, to make sense of the participants’ experiences, whilst the participants were also trying to make sense of their own experiences. An idiographic, analytical, and reflexive practice was adopted throughout the process to ensure results are representative of the experiences of the adolescents in this study. Although the use of a double hermeneutic process is a strength of the analysis, it is important to acknowledge that there may also be other interpretations of the data from a different researcher perspective [33]. Similarly, the homogeneity of the sample also means that participants’ experiences may be specific to their situation; for example, those who have not been diagnosed or sought treatment may have very different experiences. All but one participant was female and although this reflects broader sex differences in the prevalence of panic disorder [5], it would be important to explore the experience of different sexes further. Within this study we defined adolescence as aged 11–18 years. However, it must be noted that adolescence can be defined using differing theoretical frameworks (e.g., biological, social) [56], anywhere between nine and 26 years of age [57]. As adolescents reach their late teens and early twenties, significant life changes are likely to occur, such as leaving full-time education, entering full time work, going into higher education, and leaving the home environment, and therefore aspects of the experience of having panic disorder may differ from the adolescents in this study.

## Conclusion

Adolescents experienced panic disorder as a debilitating cycle of intense, physical sensations and catastrophic thoughts that ultimately overwhelmed them. This experience was consistent with the adult cognitive model of panic, as catastrophic thoughts and misinterpretation of bodily sensations led to increasing anxiety, avoidance, and safety behaviours, creating a vicious cycle. Social worries, feeling broadly misunderstood, and unhelpful responses from others, contributed to feelings of being different or abnormal and were connected to a negative self-concept. Given the significant distress experienced by adolescents with panic disorder, accessing timely and effective psychological treatment is critical. There is also a clear need for increased awareness in schools among staff and young people, to ensure that adolescents experiencing panic attacks in the school environment are well supported.

## Supplementary Information

**Additional file 1. Figure S1.** Interview schedule. Interview topic guide used in semi-structured interviews. **Figure S2.** Measures. A description of the diagnostic measures used to establish a diagnosis of panic disorder in trial participants.

## Data Availability

The research materials can be accessed by contacting the corresponding author.

## Abbreviations

IPA: Interpretive phenomenological analysis
UREC: University of Reading Ethics Committee
NHS: National Health Service
NIHR: National Institute of Health Research
AnDY: Anxiety and Depression in Young People Clinic
ADIS-C/P: The anxiety disorders interview schedule
K-SADS: Kiddie schedule for affective disorders and schizophrenia
PDSS: Panic disorder severity scale
DSM-5: Diagnostic and statistical manual of mental disorders
GP: General practitioner
