# Supplementary material for: Adolescents’ lived experience of panic disorder: an interpretative phenomenological analysis
Source: BMC Psychol. 2022 Jun 6;10:143. doi: 10.1186/s40359-022-00849-x (PMC9167912; doi:10.1186/s40359-022-00849-x)
Supplement: Supplementary file 1 — Additional file 1. Figure S1. Interview schedule. Interview topic guide used in semi-structured interviews. Figure S2. Measures. A description of the diagnostic measures used to establish a diagnosis of panic disorder in trial participants. [file 40359_2022_849_MOESM1_ESM.docx]

**Supplementary materials**

Figure S1. Interview schedule

1. **Tell me about what it’s like to have a panic attack.**

*Prompts:* situation, physical experience/ symptoms, feelings, thoughts, how was it managed, ability to talk about it. First attack, subsequent attacks.

1. **What do you think causes your panic attacks?**

*Prompts:* awareness of causes, change in cause over time, trigger situations.

1. **How has panic affected your life?**

*Prompts:* relationships, family, social, school, change in behaviour/routines, future dreams and aspirations, limitations.

1. **What have you found that helps you? What makes it worse?**

*Prompts:* coping strategies, support, in the moment/ bigger picture, getting help/treatment/diagnosis.

1. **What would you like to say to others about panic?**

*Prompts:* friends, family, teachers, health professionals, government/public, anything else you’d like to say.

Figure S2. Measures

***Anxiety Disorders Interview Schedule – child and parent version (ADIS-C/P)***

Adolescents’ diagnoses were determined using the ADIS-C/P (1). This is a structured interview, with good psychometric properties (2), designed to assess for current DSM-IV anxiety disorders and common comorbid disorders. If the adolescent met symptom criteria for a diagnosis, on the basis of his/her report or that of his/her parent, the assessor assigned a Clinician Severity Rating (CSR), ranging from 0 (absent or none), 4 (moderate) to 8 (very severely disturbing/disabling). To meet diagnostic criteria, the adolescent must have a CSR of 4 or more. The diagnosis with the highest CSR was classed as the primary diagnosis.

***Kiddie Schedule for Affective Disorders and Schizophrenia – child and parent version (KSADS-C/P)***

KSADS-C/P (3) is a structured diagnostic interview for DSM-IV affective disorders and schizophrenia was used to determine adolescents’ mood disorder diagnoses and has well established psychometric properties (3). The relevant depression and mania supplements were used. Interviews were conducted with adolescents and parents separately, and diagnoses were based on information from both interviews. Diagnosis is assessed on the presence of absence of disorders and does not include a measure of severity.

***The Panic Disorder Severity Scale for Children and Adolescents (PDSS)***

The Panic Disorder Severity Scale for Children and Adolescents (4) was administered to assess change in the frequency and severity of adolescents’ panic disorder symptoms and anticipatory anxiety and associated agoraphobia, avoidance, fear, work and social impairments. There are seven items; each rated on a 0-4 scale, with a higher score indicated greater severity with the highest total score being 28. When used in adults a cut off score of 5 or less is used to determine remission from panic disorder (5), however there is no cut off established for adolescents. It has been shown to have good psychometric properties with an adolescent population (5).

**References**

1. Silverman W, Albano A. Anxiety Disorders Interview Schedule (ADIS-IV) Child and Parent Schedules. Oxford University Press, New York; 2004.

2. Silverman WK, Saavedra LM, Pina AA. Test-retest reliability of anxiety symptoms and diagnoses with the anxiety disorders interview schedule for DSM-IV: Child and parent versions. J Am Acad Child Adolesc Psychiatry. 2001;40:937-44.

3. Kaufman J, Birmaher B, Brent D, Rao U, Flynn C, Moreci P, et al. Schedule for affective disorders and schizophrenia for school-age children-present and lifetime version (K-SADS-PL): initial reliability and validity data. Journal of the American Academy of Child & Adolescent Psychiatry. 1997;36(7):980-8.

4. Elkins RM, Gallo KP, Pincus DB, Comer JS. Moderators of intensive cognitive behavioral therapy for adolescent panic disorder: the roles of fear and avoidance. Child and Adolescent Mental Health. 2015:n/a-n/a.

5. Furukawa TA, Katherine Shear M, Barlow DH, Gorman JM, Woods SW, Money R, et al. Evidence‐based guidelines for interpretation of the Panic Disorder Severity Scale. Depression and anxiety. 2009;26(10):922-9.
